# Extremely preterm children exhibit altered cortical thickness in language areas

**DOI:** 10.1038/s41598-020-67662-7

**Published:** 2020-07-02

**Authors:** Maria E. Barnes-Davis, Brady J. Williamson, Stephanie L. Merhar, Scott K. Holland, Darren S. Kadis

**Affiliations:** 1 Department of Pediatrics, University of Cincinnati College of Medicine, Cincinnati, USA; 2 Perinatal Institute, Cincinnati Children’s Hospital Medical Center, Cincinnati, USA; 3 Department of Radiology, University of Cincinnati, Cincinnati, USA; 4 Medpace Imaging Core Laboratory, Medpace Inc, Cincinnati, USA; 5 Department of Physics, University of Cincinnati, Cincinnati, USA; 6 Neurosciences and Mental Health, Hospital for Sick Children, Toronto, Canada; 7 Department of Physiology, University of Toronto, Toronto, Canada

**Keywords:** Magnetic resonance imaging, Neonatology, Preterm birth, Brain imaging, Language

## Abstract

Children born extremely preterm (< 28 weeks gestation, EPT) are at increased risk for language and other neurocognitive deficits compared to term controls (TC). Prior studies have reported both increases and decreases in cortical thickness in EPT across the cerebrum. These studies have not formally normalized for intracranial volume (ICV), which is especially important as EPT children often have smaller stature, head size, and ICV. We previously reported increased interhemispheric functional and structural connectivity in a well-controlled group of school-aged EPT children with no known brain injury or neurological deficits. Functional and structural hyperconnectivity between left and right temporoparietal regions was positively related with language scores in EPT, which may be reflected in measures of cortical thickness. To characterize possible language network cortical thickness effects, 15 EPT children and 15 TC underwent standardized assessments of language and structural magnetic resonance imaging at 4 to 6 years of age. Images were subjected to volumetric and cortical thickness analyses using FreeSurfer. Whole-brain analyses of cortical thickness were conducted both with and without normalization by ICV. Non-normalized results showed thinner temporal cortex for EPT, while ICV-normalized results showed thicker cortical regions in the right temporal lobe (FDRq = 0.05). Only ICV-normalized results were significantly related to language scores, with right temporal cortical thickness being positively correlated with performance.

## Introduction

Extremely preterm birth (EPT, less than 28 weeks gestation) confers risk of cognitive impairment mediated by a number of risk factors, such as infection, inflammation, duration of respiratory support, duration of parenteral nutrition, and overt brain injury.^1–3^ However, less is known about factors that might confer resiliency. A subset of patients born extremely preterm do well, performing within their grade level in school with no overt deficits.^4^ Studies specifically investigating brain-based markers of resiliency that might mediate these neurodevelopmental problems are lacking. Neurodevelopmental impairment associated with prematurity includes sensory impairment, intellectual disability, and deficits in cognitive, motor, and language performance.^5–11^ Of these, language outcomes have been especially difficult to predict, with term equivalent imaging and early language testing leaving much of the variance in later performance unexplained.^7,12,13^ These impairments persist even after controlling for socioeconomic factors such as maternal education.^14–16^ Language development is of particular interest due to its close relationship with scholastic attainments and due to its contribution to peer and caregiver relationships and subsequent quality of life.^12,17–19^

### Cortical thickness and language

The cerebral cortex is composed of radial columns of pyramidal cells and interneurons and their associated projections which comprise (in most regions) the 6 layers, or lamina, of the neocortex.^20^ The cells of the cerebral cortex are not generated in the mantle but instead migrate to their terminal locations from transient proliferative zones during the course of fetal development. Neurogenesis in these proliferative zones occurs from approximately 10–25 weeks, and animal experiments suggest that an increase in this neurogenesis results in increased surface area without a significant increase in cortical thickness.^20–22^ However, destruction of neural progenitor cells at later stages results in a decrease in cortical thickness.^20^ Changes in surface area and cortical thickness can occur differentially in a region and independently of other cortical areas. Cortical thickness is related to the population of neurons in the cortical columns of the grey matter, with thickness increasing as neuronal populations grow prenatally and postnatally until pubertal years (peaks differ by region and by sex, with peaks at 7–10 years for girls and 9–11 years for boys) when subsequent pruning of synapses occurs throughout adulthood, presumed to reflect increased efficiency of cognitive networks.^23,24^ With regard to clinical status, thicker cortex versus term controls (TC) has been attributed to relative immaturity reflecting a delay in this pruning.^25^ However, recent studies have noted that it could also represent a disease state due to decreased or aberrant myelination of surrounding white matter which is registered as gray matter voxels on structural MRI.^23,26^ Decreased cortical thickness in the context of neurological disease relative to healthy controls has been theorized as a consequence of underlying axonal and neuronal damage with loss of cortical neurons, contributing to the controversy over whether thicker or thinner cerebral cortex is more advantageous in neurodevelopment.^27–29^

Cortical thickness in bilateral temporal areas has been related to reading and language function in term-born children and adolescents. Decreased cortical thickness in these areas has been found in children with reading difficulty and in children with known genetic mutations predisposing them to dyslexia and language impairment.^30,31^ Increased cortical thickness in these areas was found in children with alleles known to be protective.^30^ In a longitudinal study following children from 5 to 11 years of age, thickening in canonical language areas (Wernicke’s and Broca’s areas) and thinning of the left parietal cortex (theorized to be due to increased volume of underlying white matter following increased myelination) were positively associated with language and cognition.^32^ Conversely, a thicker Wernicke’s area has been reported in 3 to 6 year old children with speech apraxia, and the cortical thickness of the region decreased with response to therapy.^33^

### Cortical thickness in prematurity

Cortical thickness aberrations have been noted in children, adolescents, and young adults born preterm versus their term controls. However, these assessments of cortical thickness in preterm children versus healthy controls have produced conflicting findings. EPT children have decreased intracranial volume (ICV), decreased grey and white matter volume, and decreased cortical surface area globally, but regional differences are variable.^25,26,34–37^ Some studies have reported no regional differences in cortical thickness between preterm children and TC, while others have reported increased cortical thickness in preterm children (possible immaturity) and/or decreased cortical thickness in preterm children (possible disease state).^25,27,29,34,35,37,38^ The most robust pattern reported to date for EPT children and adults includes decreased thickness in bilateral temporoparietal areas and increased thickness in fronto-occipital areas.^26,28,34,35,38^ In a series of publications following the same cohort of preterm children born in Norway at 8, 15, 20, and 26 years, authors report that–while cortical thickness is decreased in the temporoparietal areas of preterm children at 8, 15, and 20 years–the developmental trajectory of cortical thinning from adolescence to young adulthood for those born preterm does not significantly diverge from their term controls.^24,26,28,34,35,39^ Of note, the majority of prior studies fail to normalize cortical thickness measurements by body mass, head size, or ICV, which could drive or modify observed group differences.

Thus, the clinical significance of atypical cortical thickness and morphology in children born extremely preterm is indeterminate. Most studies investigating the relationship between cortical thickness and language scores in preterm children have reported findings that do not survive correction for multiple comparisons.^27,35^ In very preterm (VPT) adolescents, longitudinal changes in cortical thickness were related to language-based executive function tasks, with cortical thickness in the right occipitofrontal gyrus being positively associated with scores and cortical thickness in the left superior parietal lobe being negatively correlated in the VPT group specifically.^38^ A recent study of VPT children at 8–16 years of age (which was normalized by ICV) found that cortical thickness in numerous areas, including frontal and prefrontal areas and the superior temporal gyrus, was inversely related to IQ, but they did not focus on language development.^40^ Collectively, the relationship of cortical thickness with observable behavior–particularly language–has not been robust. Conflicting reports make the utility of this measure unclear. Furthermore, conflicting hypotheses exist in the literature regarding the relationship between thickness of cortical grey matter and integrity of the underlying white matter; primarily, whether cortical thinning is the result of damage to underlying axons or a result of increasing myelination of underlying axons.^26,29,32^

Some of these discrepancies in the literature could be due to differences in clinical populations, due to changes in the era of neonatal intensive care in which the children were born, or due to methodological issues. Normalization is one such methodological issue. Published studies of cortical thickness in EPT children and infants have not reported formal normalization for ICV, which is especially important as EPT children often have smaller stature, smaller head size, and lower ICV.^25,28,29,35^ Normalizing for ICV permits assessment of focal effects (relative increases and decreases) that may be masked or even apparently reversed if we fail to account for global effects. That is, some regions that appear significantly thicker or thinner in non-normalized analysis may show effects in the opposite direction with normalization because normalization provides scaling while consistency within a group reduces variance. Some studies including EPT children have included age as a nuisance variable in analyses.^27–29,38,41^ While age and ICV are often related, they should not be assumed to be congruent, particularly in a clinical population at risk for in utero and ex utero growth restriction and growth failure.

### Theoretical model and hypotheses

Recently, we reported that young children aged 4–6 years who were born extremely preterm–but without overt brain injury or neurological deficits–had functional and effective interhemispheric hyperconnectivity between bilateral temporoparietal areas supporting language function, as indexed by magnetoencephalography (MEG).^42^ We subsequently interrogated the structural connectivity of the language network utilizing diffusion imaging, and found increased connectivity in an extracallosal pathway involving the cerebellum which was positively correlated with performance in EPT.^43^ In this current study, we investigate cortical thickness differences between these well-performing EPT children and their term controls (TC) on the whole brain level and within the previously defined language network, relating them to our prior multimodal findings. Our theoretical model is that extreme prematurity results in atypical neuronal development, and that high-performing extremely preterm children are able to perform comparably to peers though adaptive mechanisms that result in alternative functional and structural networks in the brain. Thus, we aim to test the following hypotheses:

1. Children born EPT will have alterations in cortical thickness compared to their term controls, including canonical areas that support language such as bilateral temporal and parietal regions.
2. Alterations in cortical thickness will be significantly related to language scores on standardized assessments. Based on our prior published work reporting atypical effective, functional, and structural connectivity involving right temporal cortex in these children, we hypothesize that increased cortical thickness in this area will be positively correlated with performance for the EPT group exclusively.

## Methods

### Participants

This is an observational study with 30 participants recruited from the greater Cincinnati area. EPT (n = 15) children were recruited from ongoing prospective studies of children born less than 28 weeks in the years of 2009 to 2012. TC (n = 15) were recruited through Cincinnati Children’s Hospital Medical Center (CCHMC) Clinical Trials Office research opportunity advertisements. Children in the EPT group were recruited from CCHMC and affiliated level 3 neonatal intensive care units (NICUs) if they were born at < 28 weeks gestation, had no grade 3–4 IVH on neonatal cranial ultrasound, and had Bayley Scales of Infant Development-III (BSID-III) scores within normal range at 2 years. Medical chart reviews were performed for preterm participants to verify clinical data (gestational age, cranial ultrasound results) obtained from the parents. For this study, known brain injury was determined by cranial ultrasound results from the NICU stay. Clinical MRI was not routinely obtained on these children. Children with cerebral palsy, seizures, migraines, history of learning or speech disability, or history of speech therapy were excluded from both groups. The study was approved by CCHMC IRB and conforms to the US Federal Policy for the Protection of Human Subjects. Children were tested in a single visit in 2015–2016. Written informed consent was obtained from parents and verbal assent was obtained from all children.

### Neuropsychological assessments

Children underwent assessment with the Peabody Picture Vocabulary Test (PPVT4);^44^ Expressive Vocabulary Test (EVT2);^45^ and Wechsler Nonverbal Scale of Ability (WNV).^46^ The EVT2 and PPVT4 were used to assess expressive and receptive vocabulary, respectively. Both the EVT2 and PPVT4 correlate highly with verbal intelligence, especially in children.^47,48^ These assessments are co-normed and the composite score based on the arithmetic mean of these two measures (EVT2 and PPVT4) provides a robust assay of gross language ability.

### Neuroimaging acquisition

A 3D-T1-weighted structural magnetic resonance image was obtained for each subject on a 3.0 T Phillips Achieva scanner with a T1 turbo field echo (TFE) sequence (TR/TE = 8.055/3.68 ms, 1.0 × 1.0 × 1.0 mm voxels, Matrix = 256 × 256 × 160).

### Neuropsychological and demographic analysis

Between group comparisons of continuous variables (age, performance on assessments) were performed using independent samples t-tests. Categorical variables (sex, race, ethnicity, household income) were compared between groups using Fisher’s exact test.

### Cortical thickness analysis

Images were subjected to volumetric and cortical thickness analyses on a whole-brain level using FreeSurfer (Version 6.0.0 available at https://surfer.nmr.mgh.harvard.edu/ running in Mac OSX operating system version 10.12.6.). Two analyses were performed: (1) whole brain between-groups differences in cortical thickness and (2) whole-brain analysis of between-groups differences in cortical thickness normalized by ICV. We assessed the relationship between cortical thickness measurements and language scores–both within and across groups–for the whole brain and for a priori defined language areas using correlations with a cluster-wise correction for multiple comparisons. The automated FreeSurfer pipeline for cortical reconstruction, segmentation, and non-linear surface-based registration are described in other publications.^49–51^ In brief, our processing included averaging of 3D T1 images, brain extraction, normalization, tessellation of the gray matter/white matter boundary, and surface deformation to optimally place the gray/white and gray/cerebrospinal fluid (CSF) borders at the location where the greatest shift in intensity defines the boundary.^49–51^ Cortical maps were then smoothed using a full-width-half-maximum Gaussian kernel of 10 mm and maps were aligned to a standardized spherical atlas space to match cortical geometry across subjects.^52^ In this automated routine, cortical thickness is calculated as the closest distance from the gray/white boundary to the gray/CSF boundary at each vertex.^49^ Registration quality for all subjects was visually assessed and confirmed to be satisfactory. No manual post-processing was necessary. This is likely due to the high quality of the T1 weighted images that were collected and the fact that FreeSurfer applies a nonlinear, surface-based registration. Between- and within-groups analyses were performed both without normalization by ICV and with normalization by ICV. ICV normalization was performed at each vertex by dividing thickness at each vertex by ICV. We believe this is the most defensible approach (versus others such as including ICV as a regressor) as ICV showed high variability across children when assessed by age, group assignment, and sex of the participants (see Supplementary Figs. 1, 2, and 3). All vertices were included in the analyses and between groups comparisons were performed using a general linear model approach (EPT vs. TC). For both the whole-brain analysis (327,684 total vertices) and the a priori defined language network regions (66,803 total vertices), correlational analyses were performed to assess relationship with language performance at each vertex. Language performance was assessed using a composite score of the PPVT and EVT standardized scores. Monte Carlo Z null simulations were run with 1,000 iterations to determine the cluster threshold for multiple comparisons correction.^53^ This was performed both for the whole brain analyses (default option in FreeSurfer) and for a priori defined language network regions. Family-wise error was controlled using the False Discovery Rate (FDR); results were considered significant if adjusted p-values were less than 0.05.

**Figure 1:**
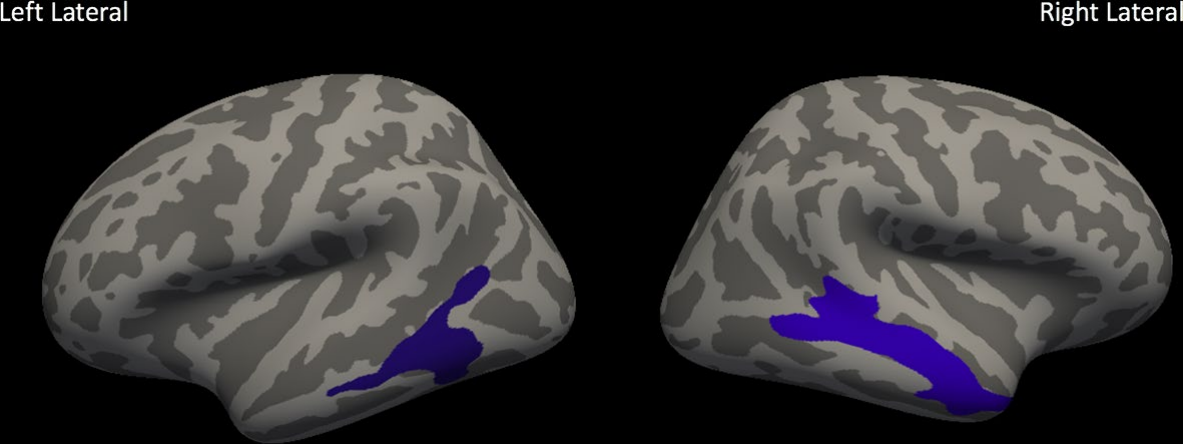
Non-normalized whole brain between groups analysis. Clusters with significantly different cortex in extremely preterm children versus term controls (FDRq < 0.05). EPT have thinner cortex in bilateral temporal areas (purple), including bilateral middle temporal gyri and the right temporal pole.

**Figure 2:**
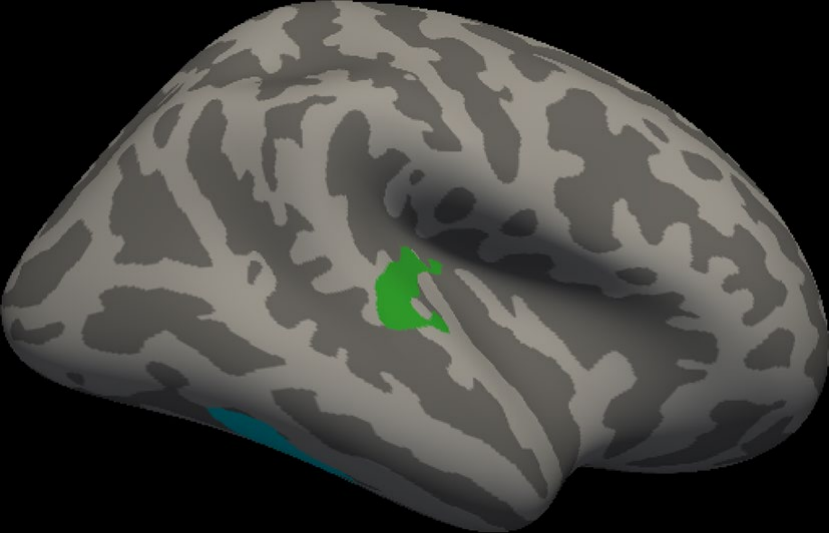
Non-normalized analysis relating to language scores for control group. Clusters with significant positive correlation with language scores for the TC group (FDRq < 0.05). Areas with significant relation to performance include the right superior temporal gyrus (green) and posterior areas of the right inferior temporal gyrus (blue).

**Figure 3:**
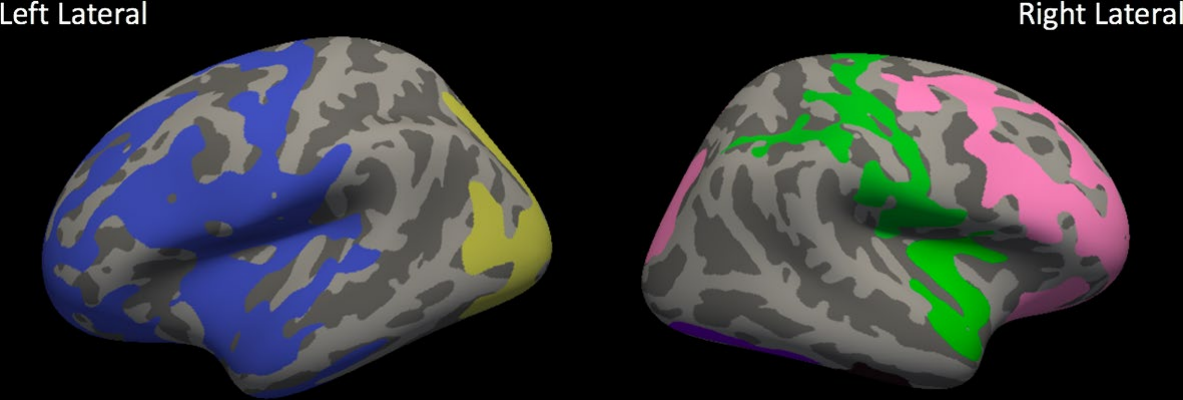
ICV-normalized whole brain between groups analysis. Clusters with significantly altered cortex in extremely preterm children versus term controls (FDRq < 0.05). When normalized for intracranial volume, EPT children have significantly increased cortical thickness, with clusters including right fronto-central areas (pink); left inferior frontal, temporal, and parietal areas (blue); left occipital areas (yellow); and right temporoparietal areas (green).

## Results

### Neuropsychological and demographic results

Demographic and neuropsychological data on this sample were previously reported.^42^ EPT (mean gestational age 26 2/7 weeks, range 24 0/7 to 26 6/7 weeks) children had no significant differences in age, sex, race, ethnicity, or family income compared to TC group. Both groups performed at or above normal limits on all assessments (mean standardized score = 100 and a standard deviation of 15). The TC group scored higher than EPT on the PPVT4, EVT2, and WNV (Table 1). The Shapiro-Wilks test for age, PPVT, EVT, and WNV were not statistically significant (*p* > 0.05) by group, indicating the assumption of normality was not violated when t tests were used to investigate between groups differences in continuous variables. The authors generated all tables and figures.

**Table 1:** Demographics and neuropsychological data for entire sample.

	Preterm (n = 15)	Term (n = 15)	*p* Value
Age (years, mean ± SD)	5.6 ± 0.9	5.7 ± 0.9	0.81
**Sex**			
Females	6	8	0.72
Males	9	7	
**Race**			
White/Caucasian	11	10	0.41
Black/African American	2	0	
Other/multiple	1	1	
No response	1	4	
**Ethnicity**			
Hispanic/Latino/Latina	0	1	0.17
Not Hispanic/Latino/Latina	14	10	
No response	1	4	
**Family income**			
< $25,000	2	0	0.33
$25,000–$75,000	8	5	
$75,000–$125,000	3	5	
> $125,000	1	1	
No response	1	4	
**Receptive language**			
PPVT-4 (Mean ± SD)	116 ± 10	135 ± 9	< 0.001
**Expressive language**			
EVT-2 (Mean ± SD)	101 ± 10	118 ± 12	< 0.001
**General abilities**			
WNV (Mean ± SD)	105 ± 14	117 ± 10	0.008

Categorical variables were tested using Fisher's Exact Test and *p* values are reported. Continuous variables were tested using t tests and *p* values are reported with 95% confidence intervals.

CI = Confidence interval. SD = Standard deviation. PPVT-4 = Peabody Picture Vocabulary Test. EVT-2 = Expressive Vocabulary Test. WNV = Wechsler Non-verbal Scale of Ability.

### Cortical thickness results: non-normalized

In the non-normalized analysis, EPT children had decreased cortical thickness in the left and right temporal areas, consistent with reports from other investigators (FDRq < 0.05, Fig. 1).^34,35,38,41^ No measures in non-normalized analyses significantly correlated with language scores for the EPT group. For the TC group, cortical thickness in right temporal areas positively correlated with language performance (FDRq < 0.05, Fig. 2). As a contrast to our a priori questions regarding cortical thickness and language, correlations with general abilities were also investigated using WNV scores. No measures in non-normalized analyses significantly correlated with WNV scores for the either group in the whole-brain analyses or for the EPT group in the a priori defined language network regions. For TC, thickness in the left inferior temporal region was positively correlated with general abilities in the network-constrained analyses (FDRq < 0.05, Supplementary Fig. 4) while non-normalized cortical thickness in the right inferior temporal region was negatively correlated with WNV performance (FDRq < 0.05, Supplementary Fig. 5).

**Figure 4:**
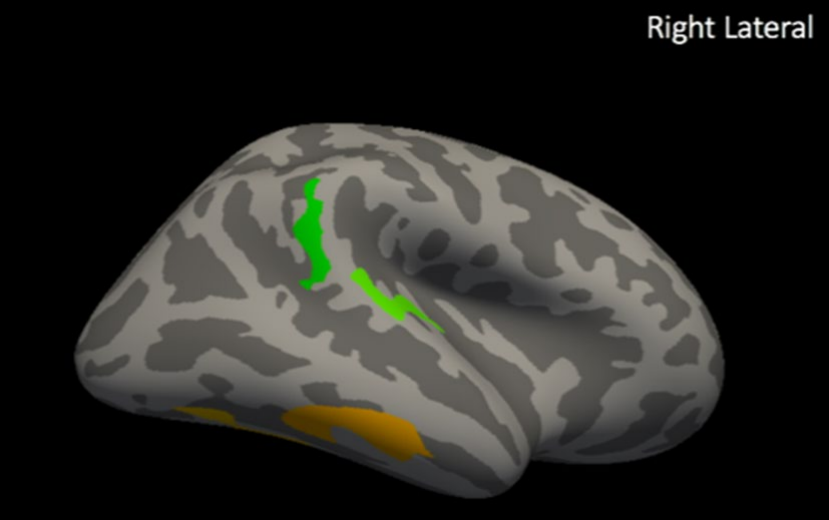
ICV-normalized analysis relating to language scores for preterm group. Clusters in which cortical thickness is significantly related to language performance for EPT children (FDRq < 0.05). When normalized for intracranial volume, cortical thickness is positively correlated with language scores for the EPT group in several clusters, including areas around the right temporoparietal junction (green) and inferior right temporal areas (orange).

**Figure 5:**
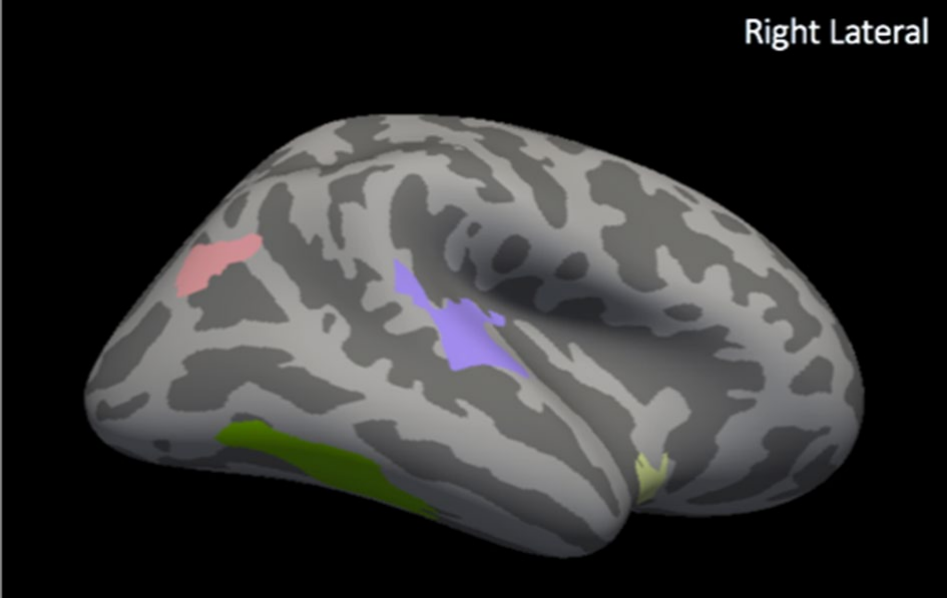
ICV-normalized analysis relating to language scores for control group. Clusters in which cortical thickness is significantly related to language performance for TC children (FDRq < 0.05). When normalized for intracranial volume, cortical thickness is positively correlated with language scores for TC in several clusters, including areas around the right temporoparietal junction (light purple); inferior right temporal areas (light green); and right parieto-occipital junction (pink).

### Cortical thickness results: ICV-normalized

In analyses normalized by ICV, EPT children had increased ICV-normalized cortical thickness relative to controls in widespread clusters of vertices, including canonical language areas in the left temporal lobe, bilateral temporoparietal areas, and bilateral frontal areas (FDRq < 0.05, Fig. 3). ICV-normalized cortical thickness in right temporoparietal areas correlated with language scores for both the EPT and TC groups (FDRq < 0.05). For the EPT group, ICV-normalized cortical thickness in the right temporoparietal junction and inferior temporal areas positively correlated with performance (FDRq < 0.05, Fig. 4). For the TC group, ICV-normalized cortical thickness in right temporoparieto-occipital areas positively correlated with performance (FDRq < 0.05, Fig. 5). As above, correlations with general abilities were also investigated. No measures in ICV-normalized analyses significantly correlated with WNV scores for the TC group in the whole-brain analyses or in the a priori defined language network regions. For the EPT group, at the whole-brain level, ICV-normalized cortical thickness in the left precentral gyrus was negatively correlated with general abilities (FDRq < 0.05, Supplementary Fig. 6). Within the a priori defined language network, normalized cortical thickness in bilateral inferior temporal areas were negatively correlated with WNV scores (FDRq < 0.05, Supplementary Fig. 7). Due to our small sample size, we could not responsibly include age and sex as distinct covariates in the model. We did, however, perform supplementary whole-brain and within-network analyses relating ICV-normalized cortical thickness to language scores while controlling for effects of age and sex, despite there being no significant differences in age or sex between EPT and controls. These results are shown in Supplementary Figs. 8 and 9 for the TC group and in Supplementary Fig. 10 for the EPT group.

## Discussion

We found that EPT children without known neurological deficit or brain injury had significantly thinner bilateral temporal cortices compared to controls in the non-normalized analysis, consistent with prior studies from other investigators. For example, a recent investigation of cortical thickness in preterm children with and without periventricular leukomalacia (PVL) found that preterm children without PVL had areas of thinner cortex versus term children (global average thickness in left hemisphere 2.87 mm for preterm versus 2.91 mm for term; average in right hemisphere 2.84 mm for preterm versus 2.88 mm for term) while those with PVL had areas of thicker cortex versus term children.^41^ Our non-normalized results in children without PVL or other overt brain injury are consistent with this report in that we found non-normalized cortical thickness was decreased versus term controls (2.85 mm versus 3.14 mm in left hemisphere for EPT and TC, respectively, and 2.82 mm versus 3.12 mm in the right hemisphere).

Our ICV-normalized analysis showed increased ICV-normalized cortical thickness bilaterally–including in canonical language areas–for EPT children. Results of the ICV-normalized analysis indicated a positive relationship between ICV-normalized cortical thickness in the right temporal lobe and language scores. This is especially interesting in light of the increased right temporoparietal functional and structural connectivity we previously reported in this sample, making this unlikely to be secondary to impaired white matter integrity in this region.^42,43^ Right temporoparieto-occipital areas are known to be rich in crossing fibers and have been previously implicated in language processes, with underlying fibers providing connections to canonical language areas.^54^ It remains to be determined whether absolute regional cortical thickness or cortical thickness normalized by ICV is a better indicator of neural functioning in a given brain region. Our findings in this sample of EPT children at school age suggest that ICV-normalized thickness is more predictive.

The current study, along with our previously reported magnetoencephalography connectivity and diffusion connectometry findings in the same cohort, could indicate that an interhemispheric pathway involving the cerebellum (a structure known to be undergoing remarkable growth during the trimester in which these children were born) is key to the development of language in extremely preterm children.^42,43,55^ In our prior reports, the degree to which EPT children exhibited this extracallosal hyperconnectivity—bypassing periventricular and callosal areas known to be especially impacted by white matter injury of prematurity—predicted language scores.^43^ This current study shows cortical thickness changes in bilateral temporoparietal regions. Collectively, findings indicate that adaptive hyperconnectivity is complemented by increased regional thickening of the cortex. This continued engagement of right hemispheric structures—contrasted to typical left lateralization of language—could reflect a beneficial adaptation in the context of preterm birth. We are currently replicating and expanding these analyses in a contemporary cohort of EPT children (with and without history of neurological findings and speech-language therapy) and term controls.

Overall brain volume is known to be highly variable but—as a whole—is decreased in preterm children and adults versus term controls. We theorize that the absolute (non-normalized) decrease in cortical thickness values as observed by our lab and other investigators using in vivo neuroimaging might reflect overall whole-brain neuronal loss due to a reduction in neuronal and glial processes generated during the fetal and perinatal period in which our EPT participants were born. This could occur as the result of known insults of prematurity, such as hypoxia, ischemia, and exposure to agents such as antenatal corticosteroids which can adversely impact neuronal development.^20,56,57^

ICV normalization accounts for individual variability in overall brain volume, thereby increasing sensitivity to identify significant focal effects in regional cortical thickness. We propose that both non-normalized and ICV-normalized measures are meaningful. In our sample, when normalized for overall ICV, we theorize that focal findings (regional thickening) might reflect an adaptive mechanism for these well-performing EPT children, whereby the extra-uterine environment to which EPT children were exposed in the NICU and in the home might drive bilateral temporal structural connectivity with resultant thickening of the right temporoparietal cortex. This theory (while speculative) and our currently reported findings are congruent with the increased bitemporal effective, functional, and structural connectivity and positive relation with language performance we have observed in this same sample of well-performing EPT children.^42,43^ Alternatively, it is possible this increased cortical thickness could indicate relative immaturity of this region (in white and/or grey matter) compared to otherwise globally decreased cortex versus term controls or be an artifact of abnormal myelination of underlying white matter.^37^ We do think the latter explanation is unlikely in light of increased structural connectivity we previously reported in this same sample of EPT children.^43^ Further studies in well-performing EPT children are needed. Our findings are unlikely to be the result of common correlates of EPT birth, such as overt brain injury or lower socioeconomic status, as children with known brain injury were excluded and no significant differences in family income (used as a proxy for socioeconomic status) were found between groups.

### Limitations

Our study specifically recruited extremely preterm children who were doing well (no known brain injury or neurological deficit). As such, these children were high-performing on neuropsychological assessments. We realize this limits generalizability of the findings. However, this did enable us to capture a relatively “pure” effect of prematurity and to start to identify markers of resiliency in this preliminary work. Our control population was also high-performing, even more so than the preterm group. The control sample was self-selected from the general community, which is a common limitation in observational cohort studies such as ours. Additionally, our sample size was small, although our a priori calculations and prior studies indicated we should have sufficient power to detect group differences for this pilot work.

### Strengths

Despite these limitations, we feel the reported experiments have several strengths. Indeed, raw metrics and ‘normalized’ metrics provide different stories; we should note that normalization (by some global measure of brain volume or head size) is common, when trying to identify ‘focal’ effects. However, more widespread (e.g., global volume decreases) can be masked with normalization alone. We felt that correcting for ICV by normalizing each vertex by ICV provided two advantages over a raw measure alone: (1) we could account for the significant group difference in overall head/brain (stature) size between groups, since all other comparisons between demographics and group and demographics and ICV yielded no significance, and (2) the normalization, as opposed to using ICV as a regressor, is preferred given the small sample size and minimizes the likelihood of overfitting. We felt that this approach was more responsible given the group differences and prior literature on ICV.

We focused on extremely preterm children who were relatively well-performing and who were without known brain injury, neurologic or psychiatric disorder, or language or learning delays. This enabled us to specifically focus on factors associated with resiliency, or positive outcomes despite the risks of prematurity. We found no significant group differences in race, ethnicity, sex, or socioeconomic status. Though our sample size was small, we did find brain markers that were positively associated with language outcome. This is promising and might enable investigators to utilize markers such as increased ICV-normalized thickness in right temporoparietal cortical regions in longitudinal prospective studies for the prediction of language outcomes in extreme prematurity. A recent study of full term and preterm neonates reported cortical thickness was significantly related to socioeconomic status and parental education.^58^ This, combined with evidence showing changes in cortical thickness in response to language therapies, suggests cortical thickness might be a unique and useful dynamic imaging marker of the response to child- and parent-targeted interventions for families of children born preterm.^33^

## Conclusions

We confirmed our first hypothesis that well-performing extremely preterm children had alterations in cortical thickness versus term controls. EPT children had decreased cortical thickness in temporal areas in non-normalized analyses. In the ICV-normalized analysis, there were widespread differences, including increased ICV-normalized cortical thickness in temporoparietal areas. We confirmed our second hypothesis that ICV-normalized cortical thickness in right temporal areas would be significantly related to language performance in preterm children, as the ICV-normalized analyses indicated increased regional cortical thickness in the right temporal lobe was significantly correlated to language scores for both groups. Our results suggest that normalized regional thickness (focal changes) are more predictive of language development in this sample of preterm children than non-normalized cortical thickness. Next steps include replication of findings in a larger cohort of EPT children, assessment of the developmental trajectory of this ICV-normalized cortical thickness, investigation of the relationship to underlying structural connectivity, and longitudinal follow-up of developmental outcomes.

## Supplementary information

Supplementary Figure Captions

Supplementary Figure 1

Supplementary Figure 2

Supplementary Figure 3

Supplementary Figure 4

Supplementary Figure 5

Supplementary Figure 6

Supplementary Figure 7

Supplementary Figure 8

Supplementary Figure 9

Supplementary Figure 10
