# Supplementary material for: Extremely preterm children exhibit altered cortical thickness in language areas
Source: Sci Rep. 2020 Jul 2;10:10824. doi: 10.1038/s41598-020-67662-7 (PMC7331674; doi:10.1038/s41598-020-67662-7)
Supplement: Supplementary file 1 — Supplementary Figure Captions [file 41598_2020_67662_MOESM1_ESM.docx]

**Full Title:**

Extremely Preterm Children Exhibit Altered Cortical Thickness in Language Areas

**Authors and Affiliations:**

Maria E. Barnes-Davis, M.D., Ph.D.^1,2*^

Brady J. Williamson, Ph.D.^3^

Stephanie L. Merhar, M.D., M.S.^1,2^

Scott K. Holland, Ph.D.^4, 5^

Darren S. Kadis, Ph.D. ^6, 7^

^1^ Cincinnati Children’s Hospital Medical Center, Perinatal Institute

^2^ University of Cincinnati, Department of Pediatrics

^3^University of Cincinnati, Department of Radiology

^4^ Medpace Imaging Core Laboratory, Medpace Inc.

^5^ University of Cincinnati, Department of Physics

^6^ Hospital for Sick Children, Neurosciences and Mental Health

^7^ University of Toronto, Department of Physiology

***Corresponding Author:**

Maria E. Barnes-Davis, MD/PhD

Assistant Professor, Department of Pediatrics

University of Cincinnati College of Medicine

Attending Neonatologist, Perinatal Institute

Cincinnati Children's Hospital Medical Center

E-mail: maria.barnes@cchmc.org

**Supplemental Figure Captions**

**Supplementary Figure 1: Plot of intracranial volume (ICV) by age for all participants.** Both groups (extremely preterm in red and term controls in blue) are represented.

**Supplementary Figure 2: Plot of intracranial volume (ICV) by group for all participants.** Boxes show the median and quartiles and violins represent the variance. Term control children are represented by dark blue and extremely preterm children (EPT) are represented by light blue. While ICV is lower on average in the EPT group, both groups have high variability.

**Supplementary Figure 3: Plot of intracranial volume (ICV) by sex for all participants.** Boxes show the median and quartiles and violins represent the variance. Male children are represented by green and female children are represented by orange. While ICV is lower on average in females there is high variability for both sexes.

**Supplementary Figure 4: Non-Normalized Analysis Positively Relating to Scores of General Abilities for Control Group within Language Network.** Clusters with significant positive correlation with scores on the Wechsler Nonverbal Scale of Ability for the TC group (FDRq<0.05). Only cortical thickness in the left inferior temporal region (represented in wine color) was positively correlated with general abilities in the network-constrained analyses.

**Supplementary Figure 5: Non-Normalized Analysis Negatively Relating to Scores of General Abilities for Control Group within Language Network.** Clusters with significant negative correlation with scores on the Wechsler Nonverbal Scale of Ability for the TC group (FDRq<0.05). Only cortical thickness in the right inferior temporal region (represented in green color) was negatively correlated with general abilities in the network-constrained analyses.

**Supplementary Figure 6: ICV-Normalized Analysis Negatively Relating to Scores of General Abilities for Preterm Group within Whole Brain.** Clusters with significant negative correlation with scores on the Wechsler Nonverbal Scale of Ability for the EPT group (FDRq<0.05). At the whole-brain level, ICV-normalized cortical thickness in the left precentral gyrus (represented in purple color) was negatively correlated with general abilities

**Supplementary Figure 7: ICV-Normalized Analysis Negatively Relating to Scores of General Abilities for Preterm Group within Language Network.** Clusters with significant negative correlation with scores on the Wechsler Nonverbal Scale of Ability for the EPT group (FDRq<0.05). Within the a priori defined language network, normalized cortical thickness in bilateral inferior temporal areas (represented in violet colors) were negatively correlated with WNV scores

**Supplementary Figure 8: Results of whole-brain analysis of performance effects within controls, normalized by ICV and controlled for age and sex.** Regions include left isthmus of the cingulate and left lateral occipital lobe. Results show a positive relationship, meaning that these regions display increased cortical thickness with greater performance.

**Supplementary Figure 9: Results of stories network analysis of performance effects within controls, normalized by ICV and controlled for age and sex.** Regions include left inferior temporal lobe, right superior temporal lobe, right inferior temporal lobe, right inferior frontal gyrus pars triangularis, and right medial prefrontal lobe. Results show a positive relationship, meaning that these regions display increased cortical thickness with greater performance.

**Supplementary Figure 10: Results of stories network analysis of performance effects within EPT, normalized by ICV and controlled for age and sex.** The right inferior temporal lobe was resolved. Results show a positive relationship, meaning that this region displays increased cortical thickness with greater performance. For the EPT group, there were no whole-brain effects passing family-wise error correction after controlling for age and sex.
