# Supplementary figures and images for: Extremely preterm children exhibit altered cortical thickness in language areas
Source: Sci Rep. 2020 Jul 2;10:10824. doi: 10.1038/s41598-020-67662-7 (PMC7331674; doi:10.1038/s41598-020-67662-7)

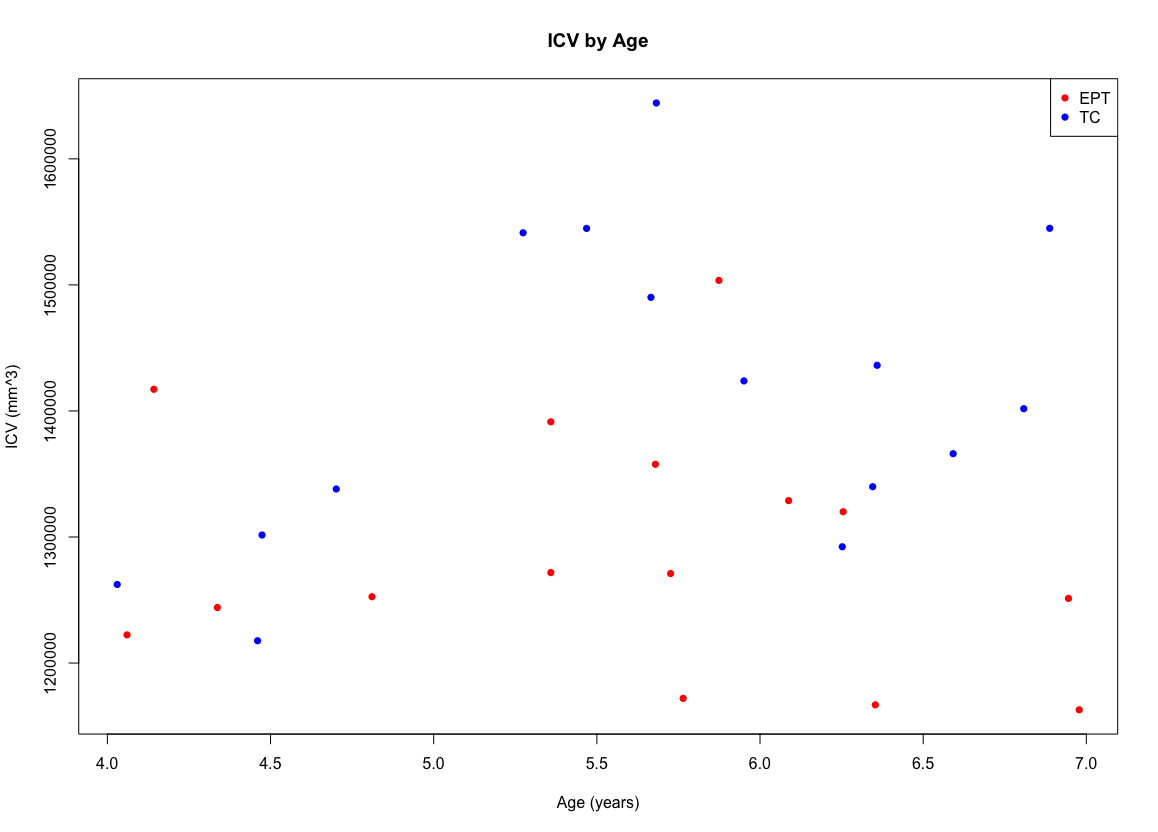

Supplement: Supplementary file 2 — Supplementary Figure 1 [file 41598_2020_67662_MOESM2_ESM.tiff]

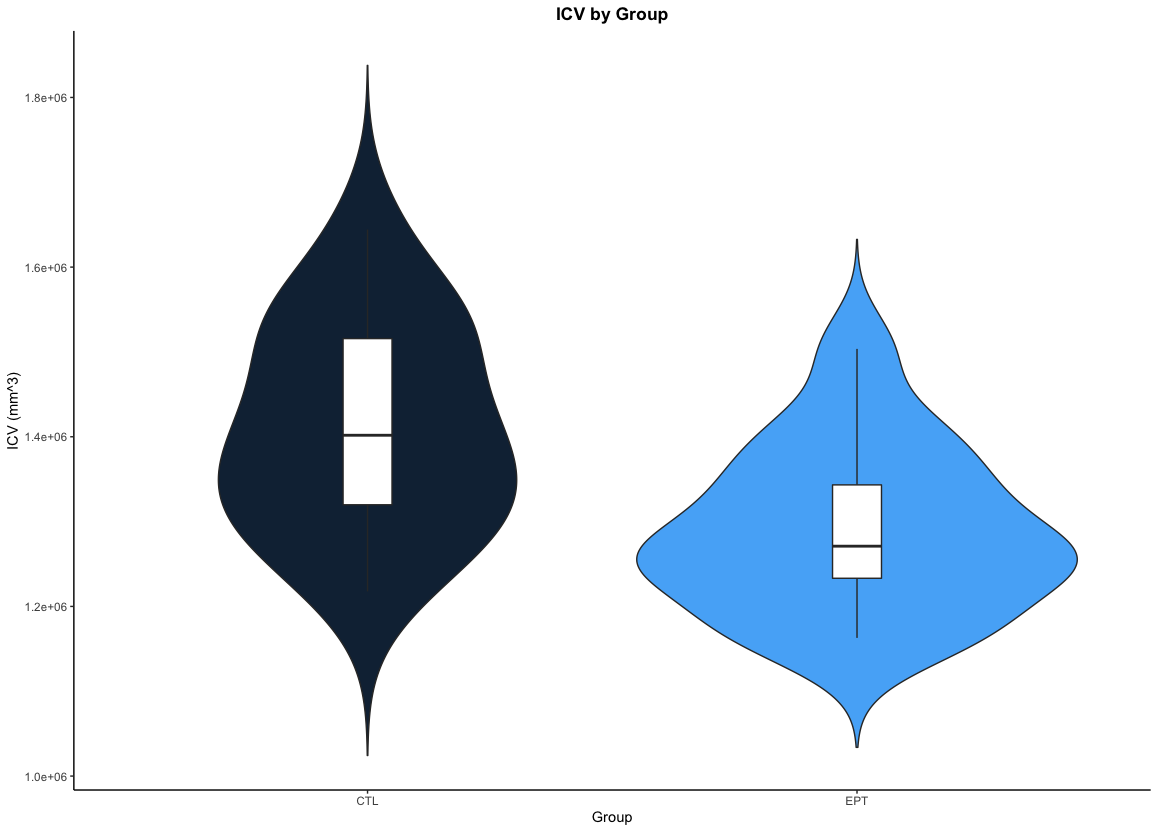

Supplement: Supplementary file 3 — Supplementary Figure 2 [file 41598_2020_67662_MOESM3_ESM.tiff]

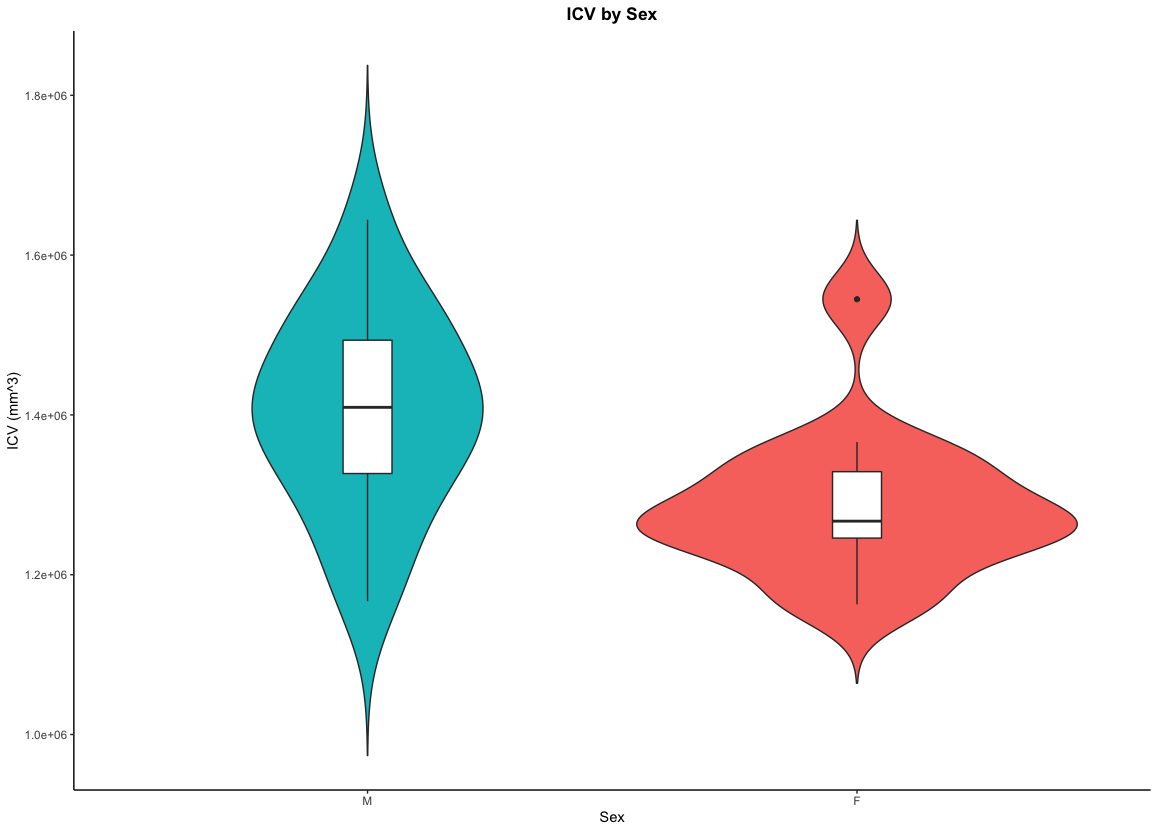

Supplement: Supplementary file 4 — Supplementary Figure 3 [file 41598_2020_67662_MOESM4_ESM.tiff]

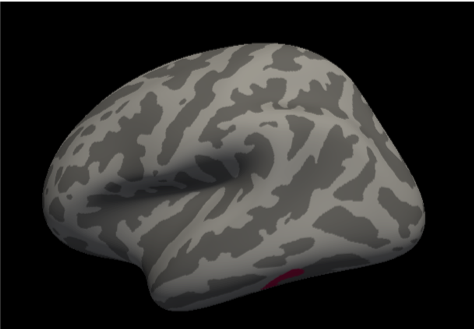

Supplement: Supplementary file 5 — Supplementary Figure 4 [file 41598_2020_67662_MOESM5_ESM.tiff]

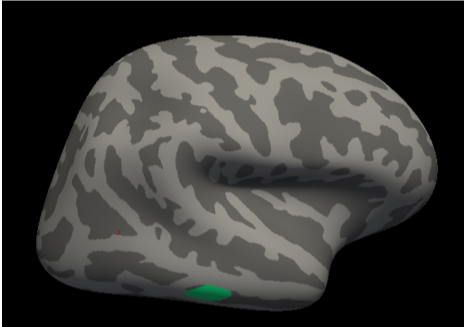

Supplement: Supplementary file 6 — Supplementary Figure 5 [file 41598_2020_67662_MOESM6_ESM.tiff]

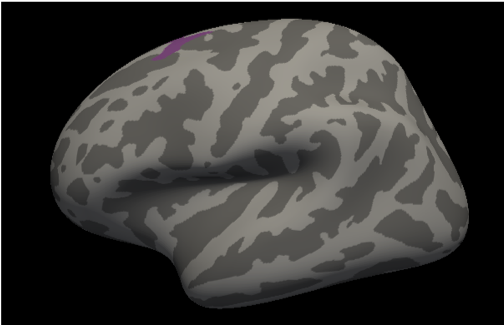

Supplement: Supplementary file 7 — Supplementary Figure 6 [file 41598_2020_67662_MOESM7_ESM.tiff]

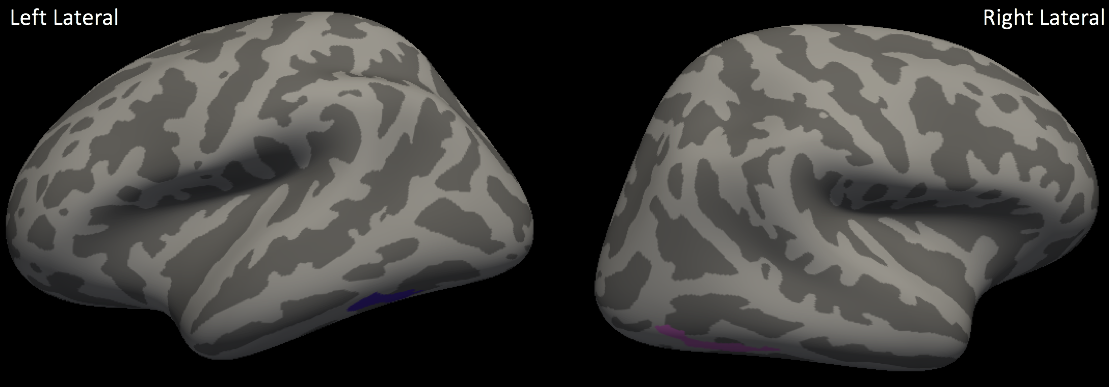

Supplement: Supplementary file 8 — Supplementary Figure 7 [file 41598_2020_67662_MOESM8_ESM.tiff]

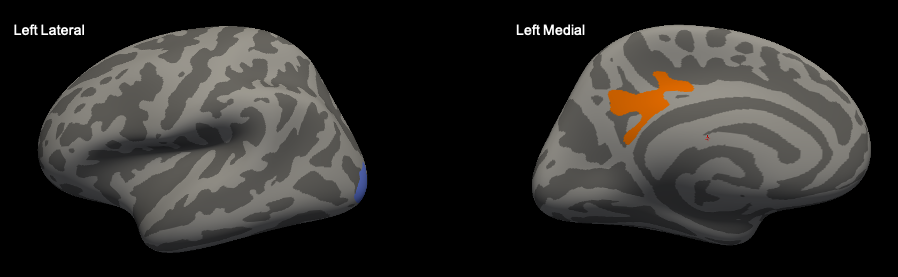

Supplement: Supplementary file 9 — Supplementary Figure 8 [file 41598_2020_67662_MOESM9_ESM.tiff]

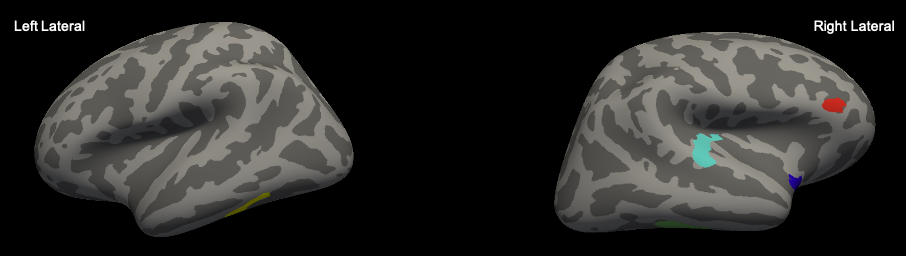

Supplement: Supplementary file 10 — Supplementary Figure 9 [file 41598_2020_67662_MOESM10_ESM.tiff]

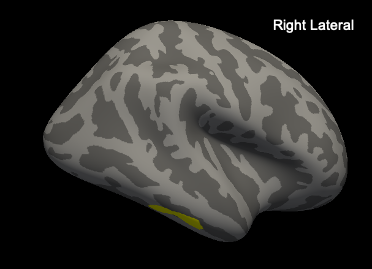

Supplement: Supplementary file 11 — Supplementary Figure 10 [file 41598_2020_67662_MOESM11_ESM.tiff]
